# Polylactide/Polycaprolactone Nanofiber Scaffold Enhances Primary Cortical Neuron Growth

**DOI:** 10.3390/polym18020294

**Published:** 2026-01-21

**Authors:** Valeriia S. Shtol, Anastasiia D. Tsareva, Kirill A. Arsentiev, Sophia P. Konovalova, Suanda A. Tlimahova, Dmitry V. Klinov, Dimitri A. Ivanov, Pavel E. Musienko

**Affiliations:** 1 Scientific Center for Genetics and Life Sciences, Sirius University of Science and Technology, 1, Olympic Ave., 354340 Sochi, Russia; shtolvaleria@gmail.com (V.S.S.); tsareva.ad@talantiuspeh.ru (A.D.T.); arsentev.ka@talantiuspeh.ru (K.A.A.); konovalova.sp@talantiuspeh.ru (S.P.K.); tlimahova.sa@talantiuspeh.ru (S.A.T.); klinov.dmitry@mail.ru (D.V.K.); 2 Institute of Translational Biomedicine, St. Petersburg State University, 199034 St. Petersburg, Russia; 3 Lopukhin Federal Research and Clinical Center of Physical-Chemical Medicine, 119435 Moscow, Russia; 4 Institut de Science des Matériaux de Mulhouse (CNRS UMR 7361), F-68057 Mulhouse, France; 5 Moscow Center for Advanced Studies, 20, Kulakova Str., 123592 Moscow, Russia

**Keywords:** cell therapy, neural tissue engineering, spinal cord injury, electrospinning, biodegradable scaffolds, porous nanofibers, poly(lactic acid), polycaprolactone, polymer matrix

## Abstract

Spinal cord injury (SCI) remains a major clinical challenge due to the limited regenerative capacity of the central nervous system (CNS). Effective scaffolds for repair must combine mechanical compatibility with host tissue, controlled degradation matching the time course of regeneration, and microarchitectural features that promote neuronal survival. Electrospun nanofibrous scaffolds mimic the structural and mechanical features of the extracellular matrix, providing critical cues for neuronal adhesion and glial modulation in neural regeneration. Here, we fabricated biodegradable poly(lactic acid)/poly(ε-caprolactone) (PLA/PCL) scaffolds using a dichloromethane/tetrahydrofuran (DCM/THF) solvent system to induce surface porosity via solvent-driven phase separation. The DCM/THF solvent system formulation produced nanofibers with porous surfaces and increased area for cell interaction. PLA/PCL scaffolds showed a Young’s modulus of ~26 MPa and sustained degradation, particularly under oxidative conditions simulating the post-injury microenvironment. In vitro, these scaffolds enhanced neuronal density up to fivefold and maintained ~80% viability over 10 days in primary neuron–glia cultures. Morphometric analysis revealed that DCM/THF-based scaffolds supported astrocytes with preserved process complexity and reduced circularity, indicative of a less reactive morphology. In contrast, scaffolds fabricated with 1,1,1,3,3,3-hexafluoro-2-propanol (HFIP) displayed reduced bioactivity and promoted morphological features associated with astrocyte reactivity, including cell rounding and process retraction. These findings demonstrate that solvent-driven control of scaffold microarchitecture is a powerful strategy to enhance neuronal integration and modulate glial morphology, positioning DCM/THF-processed PLA/PCL scaffolds as a promising platform for CNS tissue engineering.

## 1. Introduction

Traumatic injuries and neurodegenerative diseases affecting the central nervous system (CNS) rank among the most debilitating medical conditions, frequently leading to lifelong disability and reduced quality of life. Spinal cord injury (SCI), in particular, results in permanent loss of sensory and motor function due to the limited regenerative potential of mature CNS tissue [1,2]. Current regenerative strategies predominantly rely on biochemical supplementation, such as neurotrophic factors, adhesion peptides, or cell transplantation, which provide only partial benefit and do not achieve robust functional recovery [3]. Consequently, there is a pressing need for therapeutic strategies that not only facilitate structural repair but also promote the functional integration of neural circuits.

Tissue engineering approaches seek to address this challenge by designing biomaterial scaffolds that can provide structural guidance, modulate the cellular microenvironment, and deliver bioactive cues to support regeneration [3,4,5,6]. Electrospun nanofibrous scaffolds are widely used in neural tissue engineering. In contrast to melt-spun fibers [7], electrospun fibers offer a significantly enhanced ability to mimic the structural and topographical features of the native extracellular matrix (ECM) [8], thereby creating a more physiologically relevant environment for neuronal growth and repair [4,7].

The selection of an appropriate biomaterial is a critical design parameter. Synthetic biodegradable polymers such as poly(lactic-co-glycolic acid) (PLGA) [9], polyethylene glycol (PEG) [9], and blends of poly(lactic acid) (PLA) [9] and poly(ε-caprolactone) (PCL) [9] have been employed in the fabrication of neural scaffolds. PLGA offers tunable degradation [10], but its acidic byproducts can cause local inflammation [11,12]. While PEG-based hydrogels provide a hydrated environment, their lack of sufficient mechanical stability often precludes their use in spinal cord applications [13]. PLA/PCL blends combine the stiffness and bioresorbability of PLA with the flexibility and slower degradation of PCL [14], enabling better mechanical compatibility and controlled degradation kinetics [15,16].

In solution electrospinning, the final fiber morphology is critically governed by the choice of solvent or solvent mixtures [17,18,19]. The use of solvent blends with differing boiling points induces phase separation during fiber formation, resulting in porous or rough fiber surface morphologies [17]. Solvent evaporation rates and residual solvent content within the fabricated material are equally pivotal factors; solvents with elevated boiling points necessitate additional elimination steps to mitigate cytotoxicity. In this study, a mixture of dichloromethane (DCM) and tetrahydrofuran (THF) was utilized, facilitating near-complete solvent evaporation throughout the electrospinning process. The low boiling point and high vapor pressure of DCM, coupled with the controlled evaporation dynamics of THF, enabled the fabrication of uniform fibers exhibiting nanoporous surfaces, corroborating findings reported in related investigations [20,21].

In addition to well-studied scaffold parameters such as fiber diameter, alignment and chemical functionalization, the nanoscale surface porosity of individual fibers is an equally important yet less explored design variable. This type of surface porosity, distinct from interfiber pore size, can substantially increase specific surface area for protein adsorption, enhance hydrophilicity and provide nanotopographical cues that regulate integrin clustering, focal adhesion formation [8] and, consequently, neuronal differentiation, axonal guidance and glial responses. While studies have shown that nanoscale surface roughness and porosity influence cell behavior, systematic investigations of solvent-induced fiber surface porosity as a controllable microstructural parameter to modulate neuronal–glial interactions and neuronal network formation in primary neuron–glia co-cultures remain limited. This represents a gap in understanding how surface microarchitecture, distinct from bulk fiber properties, contributes to neural regeneration [22,23,24,25,26,27].

For clinical translation, scaffolds for SCI must satisfy stringent design criteria. The elastic modulus of CNS tissues reported in the literature ranges between 40 Pa and 20 kPa, depending on species, anatomical region, and testing modality [28]. In addition, scaffold degradation should proceed over 2 to 6 months, which corresponds to the typical timeline for axonal regrowth and remyelination after SCI [29,30,31,32]. Critically, degradation products should be non-toxic. For example, lactate from PLA hydrolysis not only fuels CNS metabolism but may also modulate immunity and promote repair [33,34,35,36]. These considerations highlight the importance of designing scaffolds with degradation kinetics aligned to regenerative timelines and degradation products that contribute positively to repair. Furthermore, scaffold design must account for cellular microenvironmental cues.

Astrocytes, key players in injury response, are highly sensitive to biophysical signals such as stiffness and surface morphology, which can influence their phenotype and secretory activity. While biochemical modulation of astrocytes is well-studied, the role of nanoscale architectural features, like fiber surface porosity, remains underexplored despite its potential to drive favorable astrocytic responses [37].

In this study, we investigate electrospun PLA/PCL scaffolds with solvent-induced surface porosity as platforms to modulate neuronal–glial interactions. By systematically varying polymer ratios and surface morphology, we link scaffold architecture to degradation, mechanics, and ultimately to neuronal survival, proliferation, and astrocytic reactivity in primary cortical co-cultures.

## 2. Materials and Methods

### 2.1. PLA and PCL Nanofibrous Scaffold Preparation

Poly(D,L-lactide) (PLA, Ingeo™ 4032D, M_w_ ≈ 110 kDa) was purchased from NatureWorks LLC (Blair, NE, USA). Poly(ε-caprolactone) (PCL, M_w_ ≈ 60.8 kDa) was obtained from BLDpharm (Shanghai, China) (Table 1). 1,1,1,3,3,3-Hexafluoro-2-propanol (HFIP, ≥99%) was purchased from Sigma–Aldrich (St. Louis, MO, USA) (Table 1). Tetrahydrofuran (THF, analytical grade) and dichloromethane (DCM, analytical grade) were supplied by EKOS-1 (Moscow, Russia) (Table 1).

**Figure 1:**
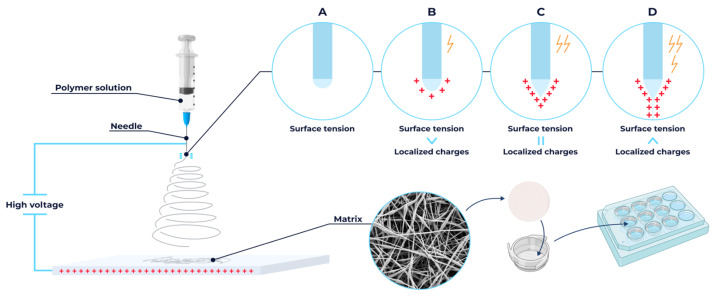
Schematic illustration of the blend electrospinning method for fabricating micro- and nanofibrous scaffolds and their subsequent preparation for cell culture applications.

Fibrous scaffolds composed of PLA, PCL, and a PLA/PCL (9:1 *w*/*w*) blend were fabricated via electrospinning system (NF-500 MECC Co., Ltd., Fukuda, Japan) (Figure 1). Polymer solutions of PLA, PCL, and PLA/PCL blends (100 mg/mL) were prepared in HFIP, DCM, and DCM/THF (8:2 *v*/*v*), respectively, and stirred for 24 h at 25 °C. After degassing, each solution was loaded into a 12 mL syringe fitted with a 22-gauge blunt-tip stainless steel needle. Electrospinning was performed onto an aluminum foil-covered plate collector under the following conditions: 25 °C, 1 mL/h feed rate, 275 mm tip-to-collector distance, and 35 kV applied voltage.

### 2.2. Morphological Analysis by Scanning Electron Microscope

The surface morphology of initial scaffolds and cell-seeded scaffolds was examined by scanning electron microscopy (SEM; Carl Zeiss Crossbeam 550, Oberkochen, Germany) using an SE2 detector. Images were acquired at 10 kV accelerating voltage and 300 pA beam current. For pristine scaffolds, samples were sputter-coated with a 10 nm Au/Pd (80:20) layer (Quorum Q150T S/E/ES Plus) to improve conductivity.

The primary mixed neuron–glia culture was prepared for SEM through sequential fixation, PBS washing, dehydration, and chemical drying. Cells were first fixed in 4% paraformaldehyde (PFA) for 30 min at room temperature, washed 3× with PBS (5 min each), and then dehydrated through a graded ethanol series (50%, 70%, 80%, 90%, 95% × 2; 10 min per step). Samples were finally treated with hexamethyldisilazane (HMDS, 99%) for 15 min at room temperature [40]. Subsequently, they were sputter-coated as described for pristine scaffolds. Fiber diameters were quantified by measuring 100 randomly selected fibers per sample using ImageJ software (version 1.54g, NIH, Bethesda, MD, USA).

Elemental analysis was performed using an energy-dispersive X-ray spectroscopy (EDS/EDX) detector (Quantax XFlash, Bruker, Oberkochen, Germany). Element distribution was mapped using Smart Map acquisition mode, enabling simultaneous X-ray data collection from each pixel within the selected area. Spectra were acquired at 10 kV acceleration voltage.

### 2.3. Quasi-Static Tensile Tests

Mechanical properties were assessed using a dynamic mechanical analyzer, DMA, (TA Instruments RSA-G2, New Castle, DE, USA) in tensile mode. Rectangular specimens (aspect ratio 1:6; thickness ≈ 90 μm) were tested at 25 °C at a constant elongation rate of 5 × 10^−3^ mm/min until fracture, without prior cyclic loading (ISO 9073-3) [41]. The evaluated parameters included the tensile modulus, determined from the initial linear region of the stress–strain curve, the ultimate tensile strength, defined as the maximum stress prior to fracture, and the elongation at break, corresponding to the strain at failure [42].

### 2.4. Internal Structure Characterization by STEM

Fiber porosity was characterized using thin-section analysis on a Carl Zeiss Crossbeam 550 scanning electron microscope (Carl Zeiss NTS GmbH, Oberkochen, Germany) equipped with a STEM detector, operated at 30 kV. For sample preparation, polymer scaffolds were embedded in Araldite resin and dried under vacuum for 2 h, followed by an additional 36 h at 60 °C. Ultrathin sections (220–270 nm) were prepared using a Leica EM UC7 ultramicrotome (Leica, Wetzlar, Germany) and mounted on 300-mesh copper grids for STEM examination.

### 2.5. Degradability

Scaffold degradation was assessed by monitoring the weight loss during a 40-day incubation period in either PBS (pH 7.4) and Fenton’s reagent, with the latter simulating oxidative stress conditions commonly associated with inflammation. The Fenton reagent consisted of 100 mM FeSO_4_·7H_2_O + 1 mM H_2_O_2_ (30% H_2_O_2_ solution) prepared in distilled water. Fresh solution (pH 3–4) was made immediately before use. All experiments were conducted at 37 °C to mimic physiological conditions. The initial dry weight of each sample (*W_i_*) was measured prior to immersion. Porous scaffold mass changes were tracked at 5, 10, 20, and 40 days (ISO 10993-13:2010) [43]. The selected exposure intervals for PLA–HFIP were 1 h, 5, 10, 20, and 40 days, with the earliest time point incorporated to assess any residual solvent present at the commencement of degradation. At each time point, scaffolds were rinsed three times with double-deionized water, dried at 37 °C for 24 h, and reweighed (*W_d_*). The weight loss was calculated using Equation (1):

(1) $$Weight\;loss\;\;(\%)\;=\frac{\;Wi-Wd}{Wi}\times \;100$$

To enable quantitative comparison of overall degradation behavior between materials, the area under the curve (AUC) of mass loss versus time was calculated. This integrated metric reflects cumulative degradation over the entire observation period and facilitates comparison between materials with different degradation kinetics.

### 2.6. Animals

All animal studies were conducted in accordance with the guidelines established by the European Community Council (Directive 2010/63/EU of 22 September 2010), and animal protocol was approved by the Ethics Committee of Sirius University of Science and Technology (protocol No. 6.2, dated 15 January 2024). 

Animals were kept in standard vivarium conditions, with free access to food and water, under controlled temperature (20–25 °C), humidity (45–70%) and 12 h light cycle. All experimental procedures were performed under aseptic and antiseptic conditions using sterile instruments.

### 2.7. Cell Culture

Fibrous scaffolds were placed in 12-well plates (Wuxi NEST Biotechnology Co., Ltd., Wuxi, Jiangsu Province, China) and submerged in 1 mL sterile PBS pH 7.2 (Figure 1). As a standard 2D control, cells were seeded on conventional tissue culture polystyrene surfaces (TCPSs). The samples were sterilized via UV-C irradiation (30 min) in a laminar air flow cabinet. After PBS removal, both the electrospun scaffolds and TCPSs were coated with 0.01% poly-L-lysine (PLL; PanEco, Moscow, Russia) to ensure comparable initial cell adhesion conditions across experimental groups. Final incubation occurred in a humidified CO_2_ incubator (37 °C, 5% CO_2_) until cell seeding.

Primary mixed neuron–glia culture was prepared from the cortex of C57Bl/6 mice pups on postnatal day 1–2 (P1–2). Following meninges removal, cortices were transferred in ice-cold Leibovitz L-15 medium (Cytiva, Wilmington, DE, USA) containing 50 µg/mL streptomycin and 50 U/mL penicillin (PanEco, Moscow, Russia). The isolated tissue was minced into small pieces using sterile microdissection scissors and digested in 0.05% trypsin-EDTA (37 °C, 10 min) (Thermo Fisher Scientific, Waltham, MA, USA). The trypsin-EDTA was removed and the tissue was washed with 10% (FBS/DMEM (Cytiva, Wilmington, DE, USA; PanEco, Moscow, Russia). DNase I (50 µg/mL) (STEMCELL Technologies, Vancouver, BC, Canada) was added to prevent cell clumping, and tissue was gently triturated using a plastic Pasteur pipette. The resulting cell suspension was filtered through a 100 μm cell strainer and centrifuged through a 4% bovine serum albumin (BSA; Proliant Biologicals, Ankeny, IA, USA) cushion (300 RCF, 7 min) to enable gentle pelleting and reduce debris contamination, as previously described for postnatal neuronal cultures [44].

Pelleted cells were resuspended in Neurobasal medium (PanEco, Moscow, Russia), supplemented with B27 (2%; PanEco, Moscow, Russia), N-2 (1%; PanEco, Moscow, Russia), GlutaMAX™ (2 mM; Gibco™, Thermo Fisher Scientific, Waltham, MA, USA), penicillin (50 U·mL^−1^), and streptomycin (50 µg·mL^−1^) (PanEco, Moscow, Russia). Cell viability and concentration were assessed using an acridine orange/propidium iodide (AO/PI) assay (Thermo Fisher Scientific, Waltham, MA, USA; Sigma–Aldrich, St. Louis, MO, USA). Cells were seeded at a density of 5 × 10^5^ cells per sample onto poly-L-lysine (PLL)-coated scaffolds. Cultures were maintained at 37 °C in a humidified atmosphere containing 5% CO_2_, with 50% of the medium replaced every 4 days.

### 2.8. Cell Viability Assessment

Cell viability on PLA, PCL, and their blend scaffolds was assessed using dual fluorescent staining with Hoechst 33342 (Invitrogen, Thermo Fisher Scientific, Waltham, MA, USA) and propidium iodide (PI; Sigma-Aldrich, Saint Louis, MO, USA). The cells were incubated with 2 µg/mL Hoechst 33342 and 2 µg/mL PI for 30 min, after which they were fixed in 4% PFA. Images were acquired using a confocal laser scanning microscope (ZEISS LSM 980 Airyscan, Oberkochen, Germany) at 10× magnification. Excitation/emission parameters: Hoechst 33342 (405 nm excitation, 445–485 nm emission, 0.5–1% laser power); PI (561 nm excitation, 600–650 nm emission, 1–2% laser power). Hoechst 33342 was used to visualize all cell nuclei, whereas PI-positive nuclei were interpreted as cells exhibiting compromised plasma membrane integrity at the time of staining. Given that fixation was applied after staining, this assay represents an endpoint assessment of relative cell viability rather than strict live/dead discrimination or cytotoxicity. Cell viability was quantified by counting live (Hoechst^+^/PI^−^) and dead (PI^+^) cells at days 1, 5, 7, and 10 using ImageJ/Fiji software V.1.54 (NIH, Bethesda, MD, USA).

### 2.9. Immunocytochemistry

Cells were fixed with 10% neutral buffered formalin for 30 min at room temperature, washed three times with PBS-T (0.05% Tween in PBS), and incubated in blocking serum (2% BSA, 1% Triton X-100 in PBS) for 60 min. Primary antibodies against NeuN (neuronal nuclear antigen; rabbit polyclonal; Invitrogen, Thermo Fisher Scientific, Waltham, MA, USA; 1:500 dilution) and GFAP (glial fibrillary acidic protein; mouse monoclonal; Invitrogen, Thermo Fisher Scientific, Waltham, MA, USA; 1:500 dilution) were applied either overnight at 4 °C (NeuN) or for 3 h at room temperature (GFAP). After washing with phosphate-buffered saline (PBS), samples were incubated for 3 h at room temperature with Alexa Fluor™ 488 goat anti-mouse IgG and Alexa Fluor™ 555 goat anti-rabbit IgG secondary antibodies (Invitrogen, Thermo Fisher Scientific, Waltham, MA, USA), both at a dilution of 1:1000 for 3 h.

Nuclei were stained with DAPI (4′,6-diamidino-2-phenylindole, Invitrogen, USA; 1:10,000) for 2 min. Samples were mounted in glycerol and imaged using a Zeiss LSM 980 Airyscan confocal microscope (10× magnification). Fluorescence images were analyzed using ImageJ/Fiji for semi-automated quantification of NeuN-positive neurons.

### 2.10. Morphometric Analysis of Astrocytes in Primary Mixed Neuron–Glia Culture

To evaluate astrocytic reactivity and morphological responses to various biomaterials, confocal z-stack images of GFAP-positive astrocytes from primary cortical cultures were analyzed. Images were pre-processed in ImageJ/Fiji to reduce background noise and enhance contrast. Maximum intensity projections were generated for subsequent analysis.

Morphometric quantification was performed using the MicrogliaMorphometry ImageJ plugin [45], which was adapted for astrocyte analysis due to the structural complexity of astrocytic processes, similar to microglial arborization. The plugin enabled semi-automated segmentation and feature extraction of individual astrocytes. A total of 1500 astrocytes across all experimental groups were analyzed.

Cell morphology was quantified using several geometric and topological parameters. The projected cell body area (*S*, µm^2^) and cell perimeter (*P*, µm) were obtained from thresholded images. Circularity (*C*) was calculated according to Equation (2), where values approaching 1 correspond to a perfect circle:

(2) $$C\;=\;\frac{4\pi S}{P^{2}}$$

The aspect ratio (cell elongation index) was derived from the ratio of the major to the minor axis of the best-fit ellipse. In addition, the arborization of astrocyte processes was characterized by the average branch length (µm), the total branch length (µm), and branchness, defined as the number of branch points in the skeletonized cell divided by the maximum cell span (Equation (3)):

(3) $$Branchness\;=\;\;\frac{Number\;of\;branch\;points\;in\;the\;cell\;skeleton}{Cell\;span}$$

The spatial organization of cells was further described using lacunarity (*L*), which quantifies the degree of space filling. It was calculated according to Equation (4), where *S* is the cell area and *S_c_* is the area of the smallest enclosing circle:

(4) $$L\;=\;\;\frac{S_{c}\;-\;S}{S}\;$$

### 2.11. Statistical Analysis

Statistical analyses were performed using GraphPad Prism v10 (GraphPad Software, San Diego, CA, USA). Data normality was assessed using the Shapiro–Wilk test, and homogeneity of variances was evaluated with Levene’s test. When both assumptions were satisfied, parametric analyses were conducted using one-way or two-way analysis of variance (ANOVA), followed by Tukey’s multiple comparisons post hoc test. If the assumption of homogeneity of variances was not met, non-parametric analysis was performed using the Kruskal–Wallis test with Dunn’s multiple comparisons post hoc test.

Degradation behavior was evaluated using time-resolved mass loss measurements, as well as an integrated parameter calculated as the area under the mass loss versus time curve (AUC), which reflects cumulative degradation over the entire observation period and enables comparison between materials with different degradation kinetics. AUC values were compared using one-way ANOVA followed by Tukey’s post hoc test. Statistical significance was defined as *p* < 0.05.

## 3. Results

### 3.1. Surface Morphology Characterization

**Figure 2:**
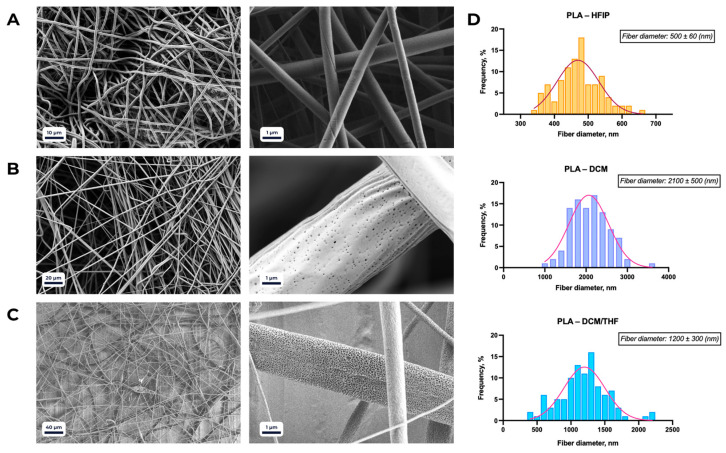
SEM micrographs of PLA-based scaffolds electrospun using different solvents: (**A**) 1,1,1,3,3,3-hexafluoro-2-propanol (HFIP); (**B**) dichloromethane (DCM); (**C**) DCM/tetrahydrofuran (THF) mixture (8:2, *v*/*v*). (**D**) Average fiber diameters (mean ± SD) corresponding to the conditions shown in panels (**A**–**C**).

Scanning electron microscopy (SEM) analysis of the fabricated scaffolds revealed a framework of randomly interwoven fibers (Figure 2). By varying the solvent and polymer composition, the surface morphology could be effectively tuned. The solvents used were HFIP, THF, and DCM.

As a control, PLA–HFIP scaffolds exhibited uniformly smooth fiber surfaces, with an average diameter of 500 ± 60 nm (Figure 2A). When DCM was used as the solvent (Figure 2B), the average fiber diameter increased to 2100 ± 500 nm, and surface irregularities became more pronounced compared to HFIP-spun fibers. Scaffolds electrospun from an 8:2 DCM/THF mixture (Figure 2C) displayed enhanced surface porosity relative to PLA/DCM samples, while maintaining fiber morphology, with an average diameter of 1200 ± 300 nm.

**Figure 3:**
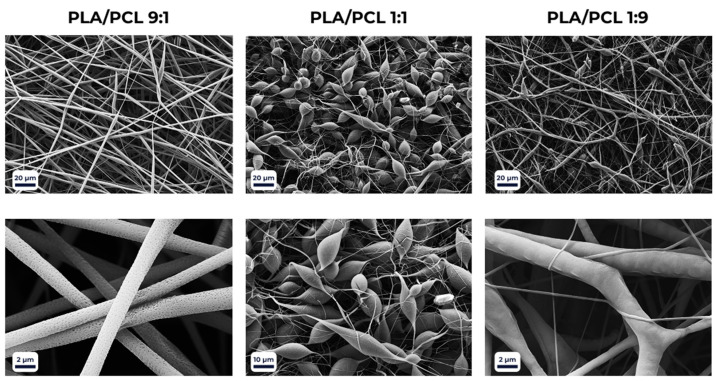
SEM micrographs of electrospun PLA/PCL blend scaffolds at different polymer ratios, illustrating the effect of composition on fiber morphology and surface porosity.

Fiber morphology was further modulated by the polymer composition of the scaffolds (Figure 3). Porous surface features were observed only in PLA-rich blends (PLA > 50%) when processed with the DCM/THF solvent system. At a PLA/PCL ratio of 1:1, the porosity disappeared, giving rise to structural irregularities such as beads, droplets, and ribbon-like fibers. Increasing the PCL fraction beyond 50% restored a more homogeneous morphology, characterized by smooth and uniform fibers.

**Figure 4:**
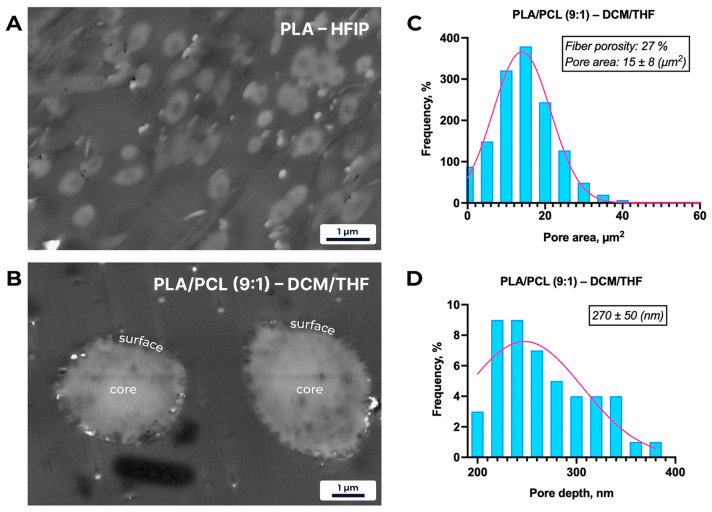
Representative STEM micrographs and pore-distribution analysis of electrospun fibers. (**A**) Cross-section of a PLA–HFIP fiber, showing a smooth, non-porous core and surface. (**B**) Cross-section of a PLA/PCL (9:1)–DCM/THF (8:2) fiber, revealing a chaotic distribution of surface-localized pores with no internal porosity. (**C**) Histogram of pore-area distribution for PLA/PCL (9:1)–DCM/THF (8:2) fibers (mean ± SD; *n* = 50 pores), demonstrating a broad range of surface pore sizes. (**D**) Histogram of pore-depth distribution for the same sample (mean ± SD; *n* = 50 pores), indicating that all pores are confined to the outer fiber layer.

STEM analysis confirmed the presence of surface-localized porosity on the fibers (Figure 4). The pores were restricted to the outer layer, with no indication of internal voids within the fiber core.

Cross-sectional micrographs of PLA–HFIP fibers (Figure 4) reveal a smooth, defect-free surface, consistent with SEM observations. In contrast, PLA/PCL (9:1) fibers electrospun from DCM/THF exhibit a heterogeneous distribution of surface-localized pores, with an average fiber porosity of 27%, a mean pore depth of 270 ± 50 nm, and an average pore area of 15 ± 8 µm^2^.

### 3.2. Mechanical Properties of Polymer Matrices

**Figure 5:**
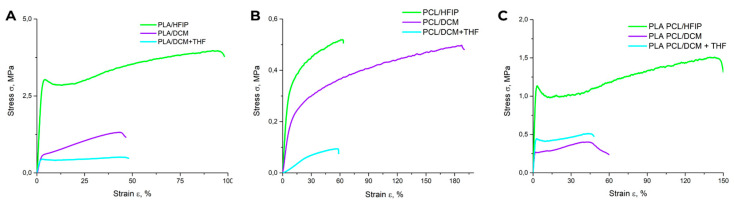
(**A**–**C**) Stress–strain curves corresponding to polymer matrices with different compositions.

Dynamic mechanical analysis (DMA) used in static tensile mode revealed marked differences in the proportional limit, elastic modulus, tensile strength, and Young’s modulus (E) across scaffolds prepared using different polymer–solvent systems (Table 2). Stress–strain (σ–ε) curves (Figure 5) showed that PLA–HFIP scaffolds exhibited the highest mechanical strength (Figure 5), a finding supported by SEM images showing defect-free fiber surfaces (Figure 2A).

For the PLA DCM and PLA DCM/THF scaffolds, both of which exhibit porous fiber surfaces, mechanical performance was markedly reduced, with elongation at break (ε) of 30 ± 3% and 45 ± 2%, respectively. The PCL-based matrices showed a further decrease in strength compared with the PLA-only group (Figure 5B), with the lowest ε (60 ± 7%) observed for the PCL DCM/THF sample.

Incorporating PCL into the polymer blend systematically lowered scaffold stiffness while enhancing elasticity. The Young’s modulus decreasing from 110 ± 30 MPa for PLA–HFIP to 26 ± 2 MPa for PLA/PCL–DCM/THF, while simultaneously enhancing ductility, as evidenced by an increase in elongation at break (50 ± 11% vs. 110 ± 18%; Table 2). Notably, the PLA/PCL–HFIP scaffold achieved the highest elongation at break (ε = 160 ± 20%), whereas the PLA/PCL–DCM and PLA/PCL–DCM/THF scaffolds exhibited reduced ε values of 50 ± 8% and 50 ± 11 %, respectively.

### 3.3. Degradability

Porous scaffolds fabricated using the DCM/THF solvent mixture were selected for degradation studies, while PLA–HFIP scaffolds with smooth fibers served as a reference. Although the PLA–HFIP samples exhibited an initial mass loss attributed to residual solvent release (1.7 ± 0.5% after 1 h in Fenton’s reagent), this loss cannot be considered indicative of polymer degradation. These values can be regarded as not caused by degradation.

**Figure 6:**
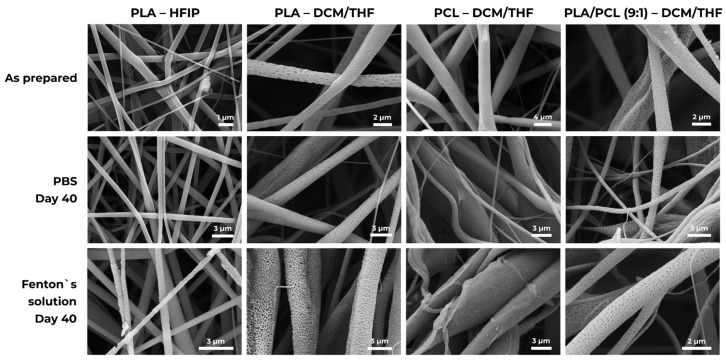
Degradation of polymer scaffolds. Representative SEM micrographs of micro- and nanofiber matrices immediately after fabrication and after 40 days’ exposure in PBS and in Fenton’s reagent.

Figure 6 presents SEM micrographs of the scaffolds before and after prolonged immersion in PBS and Fenton’s reagent. In PBS, PLA/PCL (9:1)–DCM/THF fibers developed pronounced surface texturing, indicating that degradation preferentially initiates at the fiber surface rather than within the core. Importantly, the surface morphological changes observed by SEM were not accompanied by a proportional mass loss, consistent with early-stage, surface-limited degradation originating at nanoporous regions.

**Figure 7:**
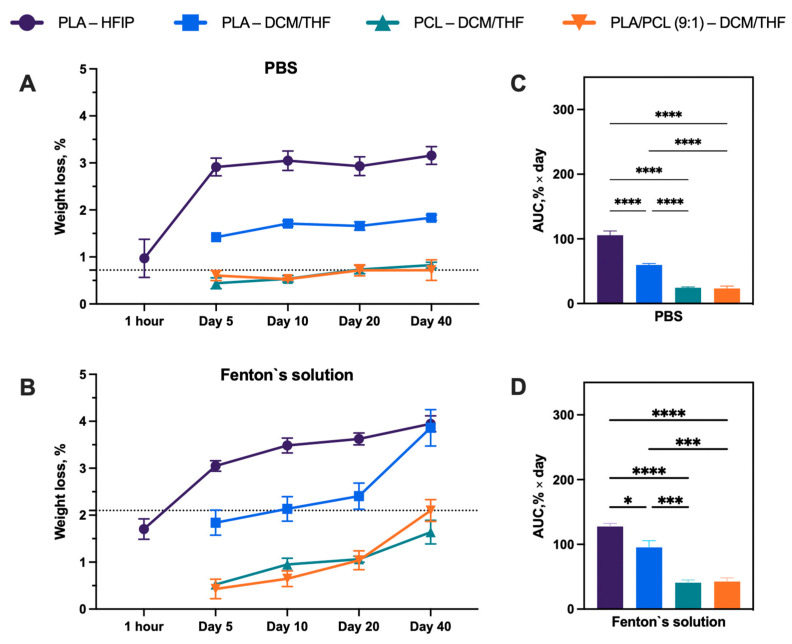
(**A**,**B**) Degradation kinetics, quantified as weight loss (%), measured over time from 1 h to 40 days. (**C**,**D**) Cumulative degradation expressed as area under the curve (AUC). Data are presented as mean ± SEM, significant differences between groups: **** *p* < 0.0001, *** *p* < 0.001, * *p* < 0.05.

Quantitative analysis of mass loss (Figure 7) demonstrated that samples incubated in Fenton’s reagent exhibited greater weight loss than those in PBS. This effect likely arises from the ability of Fenton’s reagent to mimic inflammatory conditions, in contrast to PBS, which serves merely as a physiological buffer. Importantly, the weight loss of the porous materials increased after day 20, relative to the control PLA–HFIP scaffolds. In Fenton’s reagent, the mass loss after 40 days was as follows: PLA–HFIP, 3.9 ± 0.4%; PLA–DCM/THF, 3.8 ± 0.9%; PCL–DCM/THF, 1.6 ± 0.6%; and PLA/PCL (9:1)–DCM/THF, 2.1 ± 0.5%. The corresponding values for PBS were: PLA–HFIP, 3.1 ± 0.4%; PLA–DCM/THF, 1.8 ± 0.1%; PCL–DCM/THF, 0.8 ± 0.1%; and PLA/PCL (9:1)–DCM/THF, 0.7 ± 0.5%.

### 3.4. Effect of Composition and Morphology of Polymer Matrices on Adhesion and Survival of Primary Cortical Neuronal Culture

**Figure 8:**
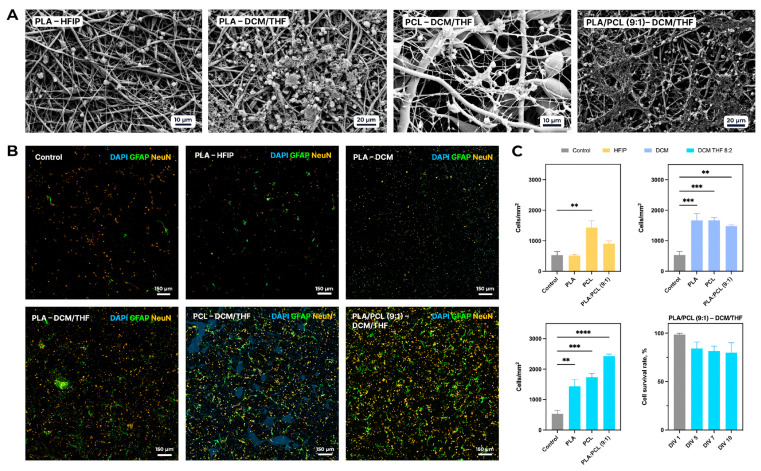
Influence of scaffold composition on adhesion, proliferation, and survival of primary mixed neuron–glia cultures from C57Bl/6 mouse cortex. (**A**) Representative SEM micrographs. (**B**) Representative immunocytochemical staining of primary cultures. (**C**) Quantitative analysis of neuronal density on each scaffold (mean ± SEM) and survival dynamics of primary mixed neuron–glia cultures over time on the PLA/PCL (9:1)–DCM/THF (8:2) scaffold (mean ± SEM). Scale bar is 150 µm. Significant differences vs. control: ** *p* < 0.01, *** *p* < 0.001, **** *p* < 0.0001.

In the case of the scaffolds fabricated from the solution in HFIP, a significant increase in neuronal density compared to the control (523 ± 258 cells/mm^2^) was observed only for the PCL sample, which exhibited 1430 ± 383 cells/mm^2^ (*p* < 0.01). All porous matrices fabricated using DCM or DCM/THF (8:2) also demonstrated significantly higher neuronal densities than the control (Figure 8A). Notably, the PLA/PCL (9:1) scaffold electrospun with DCM/THF (8:2) showed the highest neuronal density, achieving 2426 ± 122 cells/mm^2^ (*p* < 0.0001 vs. control).

As shown in Figure 8C, the viability of primary mixed neuron–glia cultures on the biodegradable PLA/PCL–DCM/THF scaffold remained consistently high throughout a 10-day cultivation period (DIV 1, 5, 7, and 10). On DIV 1, cell viability was 98.1 ± 1.3%, indicating efficient initial adhesion to the scaffold surface. By DIV 5, viability decreased to 83.5 ± 12.5%, although this reduction was not statistically significant compared to DIV 1. At later time points, viability remained stable at 81.5 ± 10.1% on DIV 7 and 79.9 ± 20.5% on DIV 10. No significant differences were observed during the entire culture period, indicating stable relative cell viability and preserved membrane integrity of neuron–glia cultures on the scaffold.

Although the same nominal seeding density was used for both 2D and 3D substrates to maintain experimental consistency, differences in effective surface area and accessible volume between these systems limit direct quantitative comparison of absolute cell densities. Therefore, comparison was interpreted primarily in terms of relative trends within each culture condition rather than direct equivalence between 2D and 3D substrates.

### 3.5. Comparative Morphometry of Astrocytes

**Figure 9:**
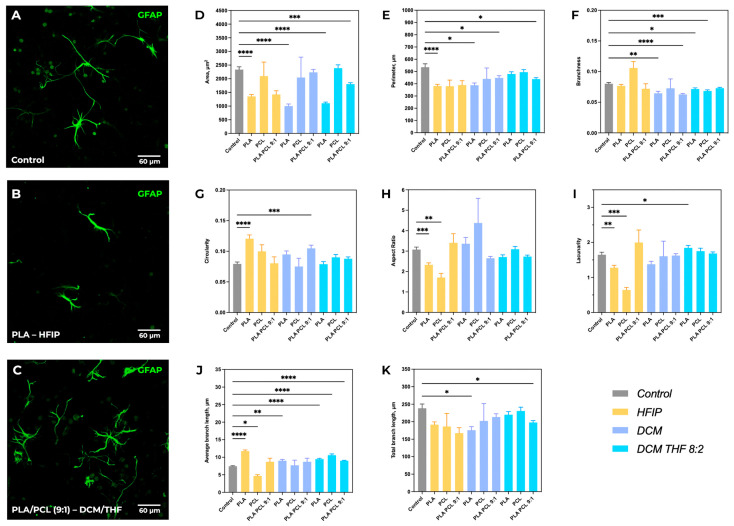
Comparative morphometric analysis of astrocytes across various scaffold compositions. Representative immunofluorescence images of GFAP-positive astrocytes cultured on control substrate (**A**), PLA–HFIP scaffolds (**B**), and PLA/PCL (9:1)–DCM/THF scaffolds (**C**). (**D**–**K**) Quantitative morphometric analysis of astrocyte morphology across all scaffold compositions. Data are presented as mean ± SEM. Significant differences vs. control: * *p* < 0.05, ** *p* < 0.01, *** *p* < 0.001, **** *p* < 0.0001.

Astrocyte morphology was found to be scaffold-dependent, with statistically significant differences observed in several morphometric parameters, including cell body area, perimeter, circularity, aspect ratio (elongation index), lacunarity, and branching characteristics of astrocytic processes (Figure 9).

The greatest reduction in astrocyte area was observed for scaffolds based on PLA–HFIP (1352 ± 839 µm^2^, *p* < 0.0001 vs. control) and PLA–DCM (1000 ± 604 µm^2^, *p* < 0.0001 vs. control), which may be associated with their higher stiffness and solvent-dependent material characteristics (Figure 9D). In contrast, scaffolds composed of PCL with added DCM/THF showed an increase in astrocyte area (2387 ± 1562 µm^2^ vs. control 2336 ± 1472 µm^2^), although this difference was not statistically significant. A significant decrease in astrocyte perimeter was recorded for PLA/PCL (9:1) in the DCM/THF blend (438 ± 227 µm vs. control 534 ± 395 µm, *p* < 0.05), indicating a simplification of cell shape and reduced complexity of their contour (Figure 9E). Area and perimeter parameters were correlated, especially in groups with the lowest values, suggesting suppression of complex astrocyte morphology development. Cell circularity significantly increased on PLA–HFIP (0.12 ± 0.07 vs. control 0.08 ± 0.06, *p* < 0.0001) and PLA/PCL–DCM (0.11 ± 0.08, *p* < 0.001) scaffolds (Figure 9G).

The aspect ratio also varied depending on the scaffold type (Figure 9H). The most pronounced decrease was observed for PLA–HFIP (2.33 ± 1.10 vs. 3.07 ± 1.78 in control, *p* < 0.001) and PCL–HFIP (1.70 ± 0.65, *p* < 0.01 vs. control). However, most materials retained an aspect ratio > 2, indicating that the elongated astrocyte morphology was generally preserved.

Lacunarity, reflecting cell distribution and the presence of intercellular spaces, decreased with the use of HFIP (PLA–HFIP: 1.30 ± 0.80, *p* < 0.01; PCL–HFIP: 1.40 ± 0.70, *p* < 0.001 vs. control) (Figure 9I). Meanwhile, lacunarity increased on PLA–DCM/THF scaffolds (1.84 ± 0.90, *p* < 0.05 vs. control).

Analysis of processes length and branching showed that the control group exhibited the highest morphological complexity (branching 0.08 ± 0.03; total process length 238 ± 177 µm) (Figure 9J,K). Significant changes in average process length were observed for HFIP-based materials: an increase on PLA–HFIP (11.76 ± 4.04 µm, *p* < 0.0001 vs. control) and a decrease on PCL–HFIP (4.67 ± 1.12 µm, *p* < 0.05 vs. control). This suggests altered astrocyte cytoskeletal organization associated with HFIP-processed scaffolds.

For porous materials (PLA–DCM), branching decreased (0.06 ± 0.03, *p* < 0.01 vs. control) while average process length increased (9.02 ± 3.04 µm, *p* < 0.05). Addition of PCL did not affect process length but reduced branching further (to 0.06 ± 0.02, *p* < 0.0001 vs. control). The use of DCM/THF solvent mixture promoted an increase in average process length, especially for PCL–DCM/THF (10.60 ± 5.53 µm, *p* < 0.0001 vs. control), but suppressed the formation of new branches.

## 4. Discussion

The key novelty of this work lies in the comprehensive demonstration that solvent-induced fiber surface porosity, rather than polymer composition alone, is a decisive parameter in directing neural cell responses. While previous studies have mainly focused on fiber alignment, diameter, or chemical functionalization, the contribution of nanoscale surface porosity to neuronal survival and astrocyte morphology has been less systematically explored. It should be noted that direct quantitative comparison of absolute cell density between 2D and 3D cultures is inherently limited by differences in effective surface area and accessible volume; therefore, the present analysis focuses on relative trends within each culture condition rather than direct equivalence. In this study, we systematically investigated how polymer composition and solvent selection influence the structural and functional performance of PLA/PCL scaffolds, with particular emphasis on solvent-induced fiber surface porosity as a design variable. Our findings demonstrate that solvent-driven microstructural changes strongly modulate both the physical properties of scaffolds and their interactions with neural cells, highlighting surface porosity as an underexplored and potentially important structural parameter in scaffold design for CNS-related applications [46,47].

Fiber morphology in electrospun scaffolds can be precisely engineered through solvent mixture composition [17,38,48]. The incorporation of solvents with disparate boiling points triggers phase separation during jet flight (needle-to-collector), yielding nanoscale surface porosity and roughness both externally and internally within fibers [17,38,48]. Critical parameters include solvent evaporation kinetics and residual solvent content, which directly influence final scaffold topography and cell–scaffold interactions. Based on these considerations, in our study, a solvent mixture of DCM/THF at a ratio of 8:2 was used, where almost complete solvent evaporation occurs during the electrospinning process. The low boiling point and high vapor pressure of DCM [20], combined with a relatively high ambient humidity of approximately 60%, enabled the formation of non-through porous fiber surfaces within the matrix. The addition of THF allows for control over the overall evaporation rate and consequently the fiber solidification speed, promoting the production of fibers with more uniform diameters. The residual amount of solvents has to be considered in the material after electroforming. Solvents with high boiling points (>100 °C) often require additional removal steps such as vacuum drying, precipitation, or sequential evaporation to minimize potential adverse biological effects [49]. In some cases, complete removal of the solvent is not always achievable due to compositional constraints. Incomplete solvent removal has been reported to negatively affect cellular responses to polymeric materials [50,51].

It has been noted that the residual solvent content in the material may be influenced by the glass transition temperature (Tg) of the polymer used [52]. Electrospinning performed below Tg results in polymer chains being in a glassy state with limited mobility, slowing solvent evaporation and leading to significant residual solvent content in the scaffold. Conversely, fiber formation at temperatures above Tg allows obtaining fibers without residual solvent presence.

The results obtained in our study are consistent with the findings of Liu et al. [21], who also employed DCM/THF mixtures (with ratios ranging from 3:1 to 1:3) to fabricate polystyrene-based scaffolds. The authors reported that this solvent system facilitates the formation of porous and rough fiber surface morphologies, confirming the versatility of the DCM/THF solvent system for different polymers.

Our study also demonstrated that the fiber morphology depends on the polymer composition of the scaffold. This phenomenon can be influenced by the phase compatibility between polymers and their concentration in solution [53]. For stable electrospinning, the polymer concentration in solution must exceed a critical threshold. Generally, fiber formation occurs when the solution reaches at least 2.5 entanglements per chain; below this value, electro-spraying of droplets takes place instead of fiber formation [54,55].

It is worth noting that the glass transition temperature Tg of PLA ranges between 55 and 60 °C [56], while the Tg of PCL is approximately −60 °C [57]. As previously described, pore formation was predominantly observed in blends containing over 50% PLA, which can be attributed to the polymer chains of PLA being in a glassy (rigid) state. In the case of PCL dissolved in a DCM/THF (8:2) mixture, pores do not form, likely due to the hydrophobicity and flexibility of PCL [58]. The absence of an azeotropic solvent ratio in this mixture (the azeotropic point for DCM/THF occurs at approximately 69/31 by mass) results in preferential evaporation of DCM during the early stages of fiber formation. Consequently, the remaining 20% THF maintains solution fluidity at a sufficient level, leading to smoothing of potential pores. For PLA-based materials, which are in a glassy state, limited chain mobility slows the evaporation of both DCM and THF, promoting pore formation on the fibers.

In the development of tissue-engineered scaffolds for implantation in the spinal cord region, a critical criterion is the consideration of mechanical properties relative to those of the native biological tissues. This matching is essential to minimize traumatic impact, maintain scaffold stability during movement, and create optimal conditions for cell growth and differentiation [59,60].

In this study, it was observed that pores in scaffolds act as structural defects, reducing the materials’ resistance to mechanical loading. Nevertheless, porous scaffolds offer advantages in tissue engineering due to their increased surface area for interaction with proteins, growth factors, and cells [61]. Consequently, such materials may effectively stimulate regeneration despite their reduced mechanical strength.

The mechanical properties of electrospun scaffolds are critically influenced by both polymer composition and solvent system, as demonstrated by our systematic evaluation of yield strength, elastic limit, ultimate tensile strength, Young’s modulus, and elongation at break (Table 2).

Samples of PLA–HFIP, PCL–HFIP, and PLA/PCL–HFIP showed substantial differences in Young’s modulus—110 ± 30 MPa, 5.6 ± 1.0 MPa, and 60 ± 7 MPa, respectively. This is attributed to PLA’s higher stiffness, which stems from its relatively high crystallinity and glass transition temperature, whereas PCL is softer and more elastic due to its lower Tg and crystallinity. Consequently, combining PLA and PCL yields materials with intermediate mechanical properties that balance the stiffness of PLA with the flexibility of PCL.

Given the highly heterogeneous and viscoelastic mechanical landscape of the spinal cord, with mechanical properties varying by orders of magnitude across tissue types, anatomical levels, and testing conditions, the concept of a single “ideal” elastic modulus for scaffold design is not realistic [62,63,64]. Consequently, scaffold design should be guided by relative mechanical compliance and functional relevance within this heterogeneous environment rather than strict numerical matching to a single reported tissue modulus. Instead, materials can be tailored for specific anatomical functions: highly porous, flexible scaffolds based on PCL–DCM may be better suited to the soft parenchyma, while stiffer, stronger compositions such as PLA/PCL–DCM/THF may serve as external structural elements mimicking the dura mater. Material stiffness not only influences mechanical matching to tissue but also affects cell behavior, including adhesion, morphology and differentiation direction [65,66].

The degradation is a critical factor for the successful in vivo application of biomaterials. Our results demonstrate that porous scaffolds fabricated using the DCM/THF solvent system exhibit markedly faster degradation kinetics compared to smooth scaffolds prepared from HFIP. This phenomenon is likely due to the increased specific surface area of the porous fiber-based materials relative to the reference samples [67,68]. The rapid initial mass loss of PLA–HFIP scaffolds within the first hour of incubation is likely attributable, in part, to residual solvent release [52], potentially comprising up to 6% of sample mass post-vacuum drying [52]. This factor should be carefully considered when interpreting degradation kinetics and distinguishing true polymer degradation from physical processes such as dehydration.

EDX analysis revealed that the as-electrospun PLA–HFIP matrices contained a substantial amount of fluorine originating from residual hexafluoroisopropanol (HFIP), reaching 3.27 ± 0.15 wt.% (Supplementary Materials, Figure S1). After vacuum drying at 37 °C for 5 days, the fluorine content decreased to 0.99 ± 0.15 wt.% (Supplementary Materials, Figure S2), indicating incomplete solvent removal. In contrast, no residual chlorine was detected in the PLA–DCM/THF scaffolds (Supplementary Materials, Figure S3).

The differences in degradation between porous and smooth scaffolds became more pronounced under Fenton’s reagent exposure. These findings are particularly relevant given that inflammatory processes, characterized by oxidative stress and elevated hydroxyl radical (•OH) activity, are typical during the early stages of spinal cord injury pathogenesis [69].

The obtained results are consistent with data reported by other researchers and demonstrate that the degradation of PLA, PCL, and PLA/PCL composite polymers corresponds to that documented in the literature [47,70]. Furthermore, it should be noted that the time frame for tissue regeneration following spinal cord injury ranges from two to six months [29,30,31]. Based on these findings, it can be concluded that the degradation of the developed materials aligns with the pace of native tissue regeneration at the injury site.

Degradation products of biomaterials, such as lactate released from hydrolyzing PLA, can significantly influence tissue repair processes. For example, hydrolytic degradation of polylactic acid (PLA) produces lactic acid, which subsequently deprotonates to lactate. Lactate, a natural metabolite of the body, plays a crucial role in the central nervous system’s energy metabolism, especially under hypoxic conditions [33] or increased neuronal activity [34]. In these states, astrocytes produce lactate via anaerobic glycolysis and transport it to neurons through monocarboxylate transporters (MCTs), providing energy and participating in the regulation of inflammatory responses [71].

Scaffolds fabricated from solutions in HFIP, characterized by smooth fiber surfaces, exhibit poor cell adhesion and low cell density, which may be related to residual solvent content and material hydrophobicity. Importantly, astrocytes on HFIP substrates exhibit a phenotype characterized by increased roundness, decreased area, and simplified branching, which may represent morphometric features previously associated with altered astrocyte activation states in the literature [72,73].

In contrast, all porous DCM and DCM/THF-based scaffolds were compared to the control group. A fivefold maximal neuronal density was achieved by adding PCL to PLA in the DCM/THF system. Astrocytes on these porous PLA/PCL–DCM/THF scaffolds exhibited morphometrics approaching the intact group with longer, more branched processes and reduced roundness, which indicated a shift toward a more physiological state of astrocytes [72]. It should be noted that GFAP immunostaining primarily reflects the organization of the intermediate filament cytoskeleton and does not fully capture fine peripheral astrocytic processes; therefore, the present morphometric analysis is limited to GFAP-positive structural features.

Future biocompatible material design should prioritize modifying scaffolds to promote neurite branching and emphasize neuronal morphometrics. Differences in cell density can be attributed to a complex interplay of factors, including scaffold morphology, hydrophilicity, and mechanical properties. As previously noted, the use of a DCM/THF solvent mixture reduces the crystallinity of the polymer blend and promotes phase separation upon PCL incorporation, thereby facilitating the formation of a highly porous structure. This porosity increases surface area for adsorption of ECM proteins such as fibronectin and laminin, which may contribute to enhanced integrin-mediated cell adhesion [74].

Porosity and fiber organization play a central role in defining the biological performance of electrospun scaffolds. A moderate packing density helps preserve pore interconnectivity, thereby facilitating nutrient and gas exchange throughout the construct [75]. At the same time, nano- to submicron-scale surface roughness and shallow pores on individual fibers may increase the effective surface area and enhance the adsorption of ECM proteins, which can indirectly support cell adhesion and spreading [76,77]. Leong et al. demonstrated that introducing surface pores below 500 nm on electrospun poly(D,L-lactide) fibers resulted in approximately 62% higher specific surface area and 80% greater protein adsorption from serum compared to smooth fibers, leading to significantly enhanced epithelial cell attachment and spreading [77]. Other studies have shown that surface chemical functionalization can influence the conformation of adsorbed proteins and thereby affect integrin-mediated recognition [76]. However, in the present study, the scaffolds were coated with poly-L-lysine (PLL), which promotes cell attachment primarily through nonspecific electrostatic interactions with negatively charged cell membranes. Therefore, while the discussed mechanisms related to ECM adsorption, RGD-mediated recognition, and focal adhesion assembly are supported by existing literature, they should be interpreted here as plausible but not experimentally verified pathways. Further studies will be required to directly elucidate the specific contribution of fiber surface nanoporosity to protein adsorption, cell adhesion mechanisms, and integrin-mediated signaling in the absence of nonspecific adhesion coatings.

## 5. Conclusions

In this study, biodegradable nanofibrous scaffolds based on PLA/PCL blends were fabricated by electrospinning from different solvent mixtures, demonstrating that both solvent composition and polymer ratio critically govern fiber morphology, mechanical behavior, and degradation kinetics. Electrospinning from a DCM/THF (8:2) mixture produced fibers with pronounced surface porosity, in contrast to the smooth morphology obtained from HFIP. The porous PLA/PCL scaffolds exhibited enhanced protein adsorption and cell adhesion, along with sustained degradation under oxidative conditions, with kinetics compatible with the regenerative time window following spinal cord injury. Their mechanical properties were also well matched to the heterogeneous mechanical environment of the CNS. Compared with smooth HFIP-derived scaffolds, porous PLA/PCL fibers displayed accelerated degradation behavior.

In vitro studies revealed that porous scaffolds supported higher neuronal survival and cell density while preserving astrocyte morphologies associated with a less reactive phenotype. In contrast, HFIP-processed scaffolds showed reduced bioactivity and cytocompatibility.

These findings highlight the importance of solvent-driven control of fiber surface porosity and polymer composition in determining scaffold performance. PLA/PCL (9:1) scaffolds fabricated using DCM/THF combine favorable mechanical properties, controlled degradation, and enhanced neural cell compatibility, positioning them as promising candidates for CNS repair. Future studies will focus on optimizing scaffold mechanics, incorporating bioactive cues, and validating regenerative efficacy in vivo.

## 6. Limitations and Future Directions

This study is limited to in vitro models using primary rodent cortical cultures. While these systems provide valuable insights into early cellular responses, they do not recapitulate the complexity of the injured spinal cord, including immune cell infiltration, vascularization, and inhibitory myelin debris. Furthermore, we did not assess intracellular signaling pathways (e.g., NF-κB, STAT3) that regulate astrocyte polarization. Furthermore, long-term degradation profiles beyond 40 days remain uncharacterized, and the biological impact of accumulated degradation byproducts such as lactate requires further investigation under physiological conditions.

Future work should focus on in vivo validation in spinal cord injury models to assess functional integration, axonal regeneration, and behavioral recovery. Additionally, surface modification with neurotrophic factors (e.g., BDNF, GDNF) could further enhance the regenerative potential of these scaffolds.

## Figures and Tables

**Table 1:** Physical properties of the solvents [38,39].

Solvent	Boiling Temperature (°C)	Electrical Conductivity at 25 °C (S m^−1^)	Surface Tension at 20 °C (mN m^−1^)	Dielectric Constant at 20 °C	Viscosity at 25 °C (cP)	Vapor Pressure at 25 °C (kPa)
HFIP	59	—	15	15.57	1.65	16
DCM	40	4.3 × 10^−11^	28.12	9.1	0.43	53
THF	66	4.5 × 10^−5^	28	7.6	0.36	19

**Table 2:** Mechanical properties of electrospun scaffolds for different polymer-solvent systems.

Material	Yield, MPa	Elastic Limit, MPa	Ultimate Tensile Strength, MPa	Young’s Modulus, MPa	ε, %
PLA–HFIP	2.7 ± 1.0	2.3 ± 0.6	3.8 ± 1.3	110 ± 30	110 ± 18
PCL–HFIP	0.40 ± 0.06	0.2 ± 0.04	0.50 ± 0.1	5.6 ± 1.0	50 ± 24
PLA/PCL–HFIP	1.0 ± 0.2	1.0 ± 0.2	1.6 ± 0.3	60 ± 7	160 ± 20
PLA–DCM	0.40 ± 0.08	0.2 ± 0.05	0.40 ± 0.04	8.5 ± 6.0	30 ± 4
PCL–DCM	0.30 ± 0.06	0.20 ± 0.02	0.50 ± 0.06	2.60 ± 0.5	190 ± 40
PLA/PCL–DCM 8:2	0.30 ± 0.06	0.20 ± 0.08	0.4 ± 0.06	20 ± 15	50 ± 8
PLA–DCM/THF 8:2–	0.60 ± 0.04	0.40 ± 0.04	1.30± 0.06	22 ± 7	45 ± 2
PCL–DCM/THF 8:2	0.100 ± 0.003	0.040 ± 0.006	0.100 ± 0.004	0.20 ± 0.02	60 ± 7
PLA/PCL 9:1–DCM/THF 8:2	0.40 ± 0.04	0.40 ± 0.03	0.50 ± 0.03	26 ± 2	50 ± 11

## Data Availability

The original contributions presented in this study are included in this article; further inquiries can be directed to the corresponding author.

## Supplementary Materials

The following supporting information can be downloaded at: https://www.mdpi.com/article/10.3390/polym18020294/s1, Figure S1. EDX analysis of residual solvent content in a freshly electrospun PLA–-HFIP polymer matrix; Figure S2. EDX analysis of residual solvent content in a PLA–-HFIP polymer matrix dried for 5 days at 37 °C under vacuum; Figure S3. EDX analysis of residual solvent in a freshly electrospun PLA–-DCM/THF polymer matrix.
