# Supplementary material for: Polylactide/Polycaprolactone Nanofiber Scaffold Enhances Primary Cortical Neuron Growth
Source: Polymers (Basel). 2026 Jan 21;18(2):294. doi: 10.3390/polym18020294 (PMC12845921; doi:10.3390/polym18020294)
Supplement: Supplementary file 1 [file polymers-18-00294-s001.zip › polymers-4069885-supplementary.pdf]

Supplementary Materials:

Polylactide/Polycaprolactone Nanofiber Scaffold Enhances Primary Cortical Neuron Growth

Valeriia S. Shtol [1,2,†], Anastasiia D. Tsareva [1,†], Kirill A. Arsentiev [1], Sophia P. Konovalova [1,2], Suanda A. Tlimahova [1], Dmitry V. Klinov [1,3], Dimitri A. Ivanov [1,4,*] and Pavel E. Musienko [1,2,5,*]

[1] Scientific Center for Genetics and Life Sciences, Sirius University of Science and Technology, 1, Olympic Ave., 354340 Sochi, Russia; shtolvaleria@gmail.com (V.S.S.); tsareva.ad@talantiuspeh.ru (A.D.T.); arsentev.ka@talantiuspeh.ru (K.A.A.); konovalova.sp@talantiuspeh.ru (S.P.K.); tlimahova.sa@talantiuspeh.ru (S.A.T.); klinov.dmitry@mail.ru (D.V.K.)

[2] Institute of Translational Biomedicine, St. Petersburg State University, 199034 St. Petersburg, Russia

[3] Lopukhin Federal Research and Clinical Center of Physical-Chemical Medicine, 119435 Moscow, Russia

[4] Institut de Science des Matériaux de Mulhouse (CNRS UMR 7361), F-68057 Mulhouse, France

[5] Moscow Center for Advanced Studies, 20, Kulakova Str., 123181 Moscow, Russia

* Correspondence: ivanov.da@talantiuspeh.ru (D.A.I.); musienko.pe@talantiuspeh.ru (P.E.M.)
† These authors contributed equally to this work.

Determination of residual solvent in electrospun scaffolds

Quantitative assessment of residual hexafluoroisopropanol (HFIP) in electrospun scaffolds was performed using EDX spectroscopy.

The electrospun PLA–HFIP scaffolds contained 3.27 ± 0.15 wt.% of fluorine pertinent to residual HFIP (Figure S1). Subsequent application of vacuum drying conditions (37°C, 5 days) resulted in a reduction in residual fluorine to 0.99 ± 0.15 wt.% (Figure S2).

PLA–DCM/THF scaffolds prepared using alternative solvents showed no detectable chlorine (Figure S3). This means that the Cl concentration was below the EDX detection limit of 0.1 wt.%.

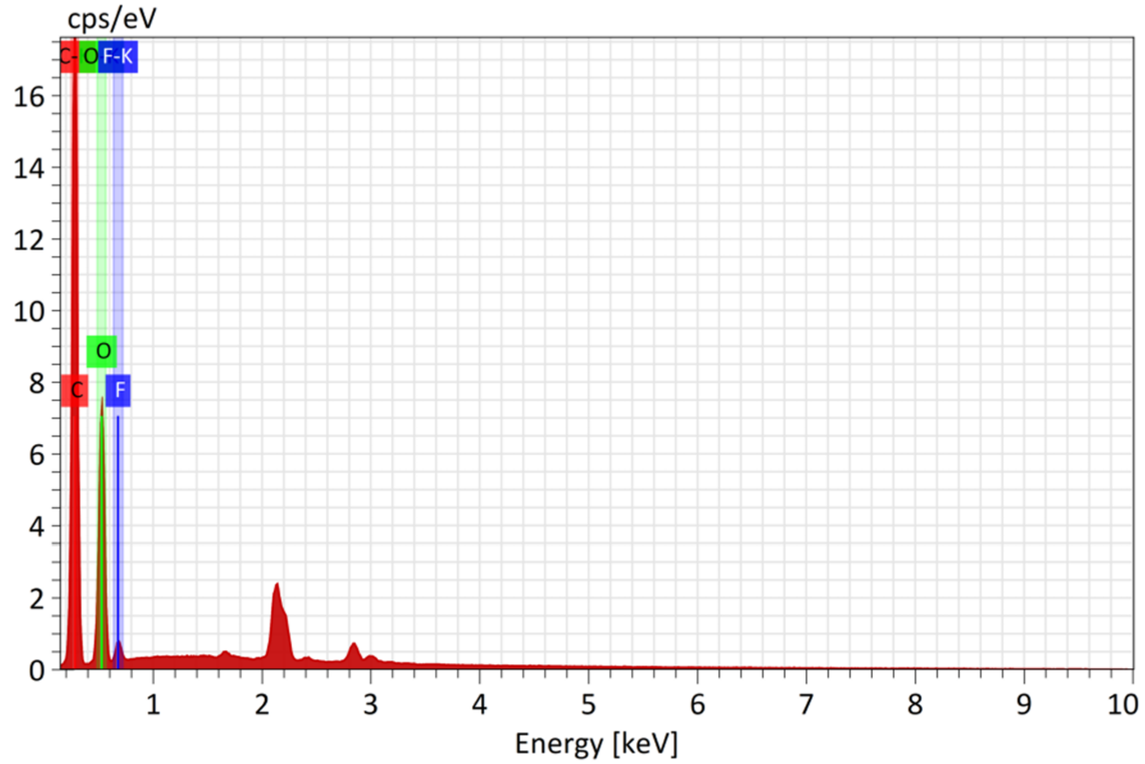

Map

Element	At. No.	Netto	Mass [%]	Mass Norm. [%]	Atom [%]	abs. error [%] (1 sigma)	rel. error [%] (1 sigma)
Carbon	6	295133	58.32	58.32	65.37	2.03	3.48
Oxygen	8	129099	38.40	38.40	32.31	1.59	4.13
Fluorine	9	11607	3.27	3.27	2.32	0.15	4.43
		**Sum**	**100.00**	**100.00**	**100.00**		

Figure S1. EDX analysis of residual solvent content in a freshly electrospun PLA–HFIP polymer matrix.

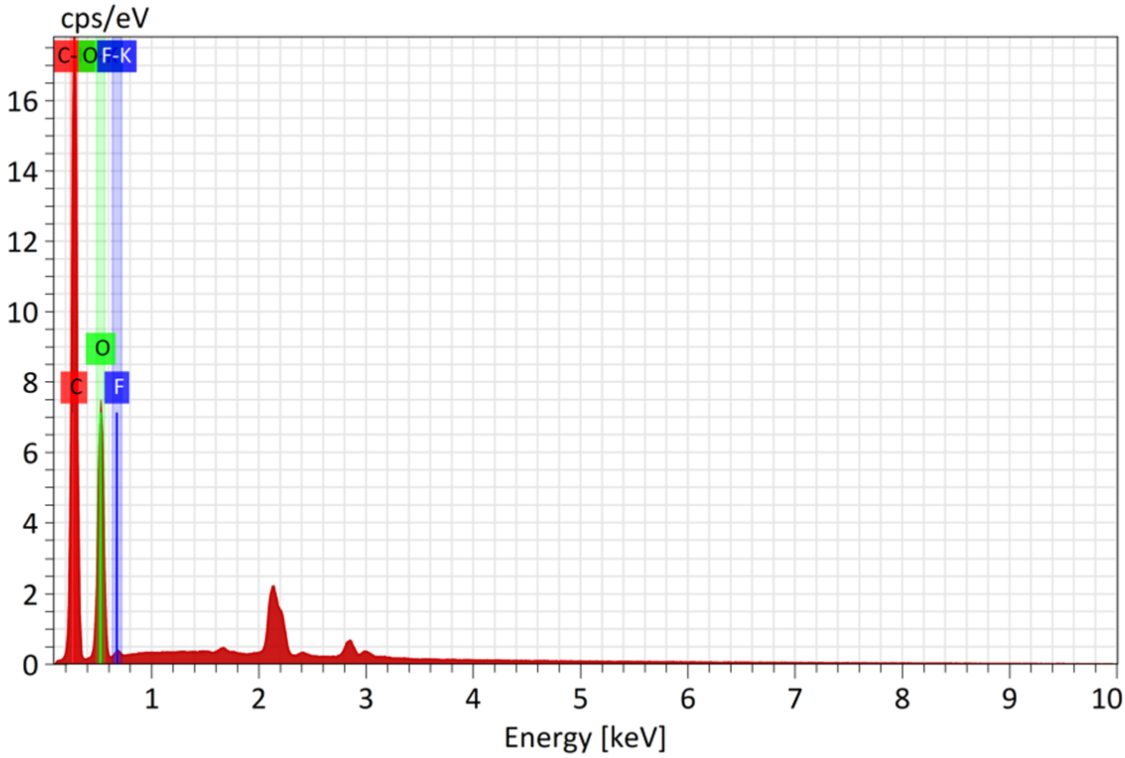

Map

Element	At. No.	Netto	Mass [%]	Mass Norm. [%]	Atom [%]	abs. error [%] (1 sigma)	rel. error [%] (1 sigma)
Carbon	6	288532	59.18	59.18	65.97	2.04	3.45
Oxygen	8	126200	39.82	39.82	33.33	1.65	4.14
Fluorine	9	3274	0.99	0.99	0.70	0.05	5.33
		**Sum**	**100.00**	**100.00**	**100.00**		

Figure S2. EDX analysis of residual solvent content in a PLA–HFIP polymer matrix dried for 5 days at 37°C under vacuum.

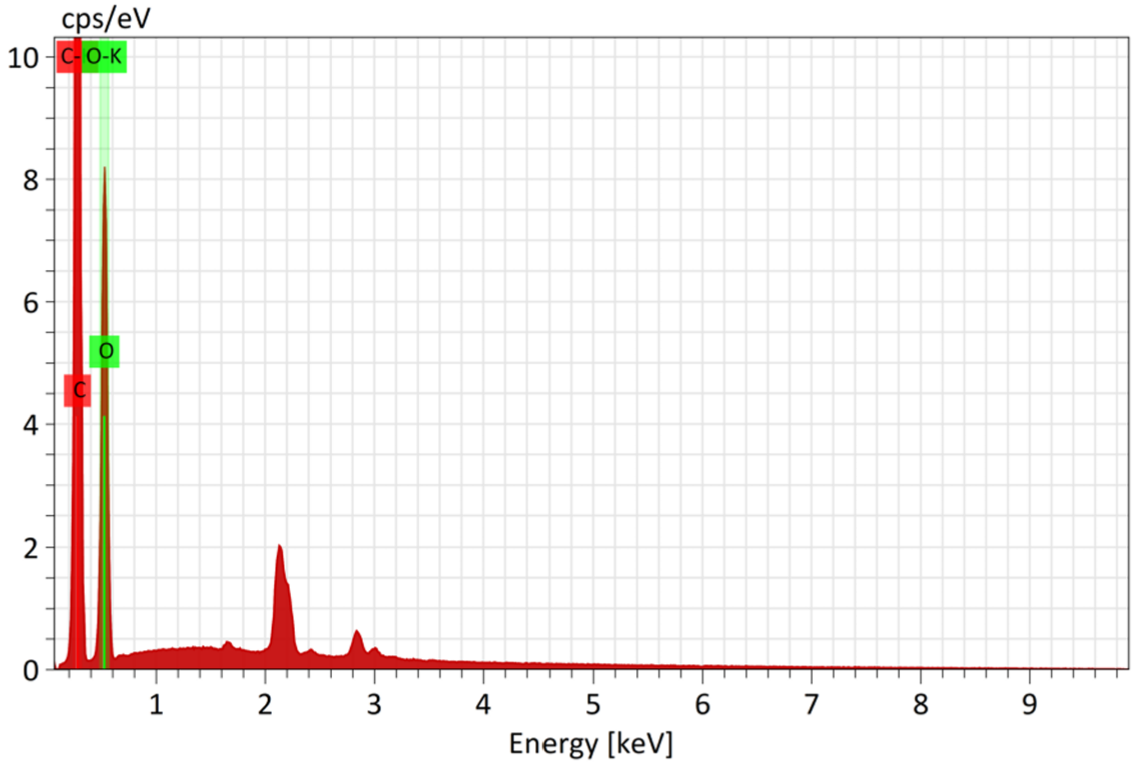

Map

Element	At. No.	Netto	Mass [%]	Mass Norm. [%]	Atom [%]	abs. error [%] (1 sigma)	rel. error [%] (1 sigma)
Carbon	6	293296	57.83	57.83	64.62	2.00	3.46
Oxygen	8	141905	42.17	42.17	35.38	1.73	4.11
		**Sum**	**100.00**	**100.00**	**100.00**		

Figure S3. EDX analysis of residual solvent in a freshly electrospun PLA–DCM/THF polymer matrix.
